# Supplementary material for: RNA binding protein Caprin-2 is a pivotal regulator of the central osmotic defense response
Source: eLife. 2015 Nov 12;4:e09656. doi: 10.7554/eLife.09656 (PMC4641828; doi:10.7554/eLife.09656)
Supplement: Supplementary file 1. — The rat Caprin-2 gene. (a) The sequence of full-length rat brain Caprin-2 cDNA. (b) The predicted amino acid sequence of full-length rat brain Caprin-2 protein. (c) Alternatively spliced isoforms of rat brain Caprin-2. (d) Hypothetical functional domains of the rat brain Caprin-2 protein based on alignment to the Xenopus RNG105 protein sequence, a paralogue of the well-analyzed rat Caprin-1 protein, which is highly homologous to Caprin-2. DOI: http://dx.doi.org/10.7554/eLife.09656.014 [file elife09656s001.docx]

**Supplementary File 1**

**RAT CAPRIN 2**

(a) cDNA cloned from rat brain and verified by sequencing.

Exons marked black and blue were predicted on the basis of GT and AG flanking regions of introns

>atgaagtcagccaagtcccaagtgaaccacactcagcaaggggaaaaccagcgggctctgagccccctgcagtctactctcagttctgctgcatctccttcccaggcatacgaaacctatattgataatggacttatatgccttaaacacaaaattaggaacatcgagaagaagaagctcaaactggaagattacaaagatcgcctgaaaaatggagagcagcttaacccagaccagttggaagcagtggaaaagtatgaagaagtacttcataatttggaatttgccaaggagcttcagaaaaccttttctgcactgagccaagatctcctgaaagcgcagaaaaaggcccagagaagggagcacatgctaaaacttgaggccgagaagaaaaagcttcgaactatacttcaaattcagtatgtattacagaacttgacacaagaacatgtacagaaagacttcaaagggggcttgaatggtgcaatgtatttgccttcaaaagaacttgactacctcattaaattctcaaaactgacctgccctgaaagaaatgaaagtttgagtgttgaagaccagatggagcagtatccttgtacttttgggaccttttggaaggtagtgagaaagcagtggtaggaacaacatacaaacatgtgaaagacctgctgtccaaattgctgcactcaggttattttgaaagtgtcccagttctcaggaattctaaggaaaaaacagaagaaatgttaatgcagtcagaaaagagaaagcagttactgaagactgagtctatcaaagagtcagaatctctgaaggaacttgtacagccagagatacagccgcaggagtttcttaacagacgctatatgacagaagtaaatttttcaagaaaacaagaaaatgaagaacaatcctgggaagcagattatgctaggaaaccaggtctcctcaaatgctggaatacacttccagaaccagatggtcaggagaagaagaaggagtccttggagtcgtgggagtcttctcttaagtctcaggaggtatccaagcctgtggtgtctttcgaacaggagaagctcaggccaacattacaggaagagcagaagcagcagatttccatggcacctgtcagtcaatggaagccagaaagccctaagtccaaagtgggcagccctcaagaagagcagaatgtacaggagacgccaaagccgtgggtggttcagccacagaaagaacaagatccaaagaagctacctcctggatcctgggcagtatctgtgcagagtgaacagagtggcagcagatcctggaccactcctgtgtgcagagaacaggcttcagtgcagcctgggactccagtatcctgggagaacaatgctgagaaccagaaacactccttagtaccacaatcacagatctctctgaagtcctggggagcagcttcagcaggcctcttaccaaatgacaaggtccctcccaggaagttaaatgtagagcccaaagatgtgcctaagcccatgcctcagcctatagactcttcctctccctttccaaaggatccagcattgaggaaagaaaaactgcaggacctcatgacccagattcaaggaacttgtaactttatgcaagagtctgttctagatgtcgacacaccctcaagtgcaattccatcttctcagccgccttcagcttcgccagtctctacagtatctgcagaacaaaacttgtccaaccaaagtgattttcttcaagagccatcaaaggcttcttctccagttacttgtagctcgaatgcttgcttggttactactgatcaggcttctctgggatctgaaacagagtttatgacctcagagacccctgagatggtggctcccccctgcaagccagcatctgcacttgcttctccaaatcctccactgtcgaagggcttccagttacctcctgcaagtgggagctcggcagccattagcacagcaccctttcaggccatgcagacagtatttaatgttaatgcacctctgcctccacggaaagaacaagcaatgaaagaatctccttattcatctggctacagtcaaagttttacttcatcaagtacacagacagtatcccaatgtcagctcccagctgtacacgtggagcagacaacccaacctcccgagactgctgcaggttaccatcctgatggaactgttcaagtaagcaatgggagccttgccttttacccagcacccacgagtatgtttcccagacctgctcagccatttatcagtagtaggggggctctgagaggatgttcacgtggagggaggttactaatgaatccttatcggtctcctggtagctacaaaggttttgatagttacagaggccttccctcagcttcaagtgggacttacagccaactgcagctgcaagctagagagtatcctgggacaccttactctcagagggataatttccagcagtgttataaaagatcagggacatctagtggtcttcaggcaaattcaagagcagggtggagcgactcctctcaggtgagcagcccagagagagacagcgagacttttaacagtggagactctggggtaggagactcccggagcatgaccccagtggatgtgccagtgacaagcccagcagccgccattctgccagtacacgtctatcctctgcctcagcaaatgcgagttgccttctcagctgccagaacatccaatctggctcctggaactttagaccaacctattgtgtttgatcttctcctgaacaacttgggagagacctttgatcttcaacttggtagattcaattgcccagtaaatggcacttacgtgttcatttttcacatgctaaagctggctgtgaatgtaccactgtatgtcaacctcatgaagaatgaggaggtcttggtgtcagcctatgccaatgatggtgctccagaccatgagacagcaagcaaccatgctattctccagctcctccagggagataagatatggttgcgcttacacaggggagcgatttatggaagtagctggaaatactctacattttcaggctatcttctttatcaagattga//

(**b**) Protein sequence, rat Caprin2, 1029aa

MKSAKSQVNHTQQGENQRALSPLQSTLSSAASPSQAYETYIDNGLICLKHKIRNIEKKKLKLEDYKDRLKNGEQLNPDQLEAVEKYEEVLHNLEFAKELQKTFSALSQDLLKAQKKAQRREHMLKLEAEKKKLRTILQIQYVLQNLTQEHVQKDFKGGLNGAMYLPSKELDYLIKFSKLTCPERNESLSVEDQMEQSSLYFWDLLEGSEKAVVGTTYKHVKDLLSKLLHSGYFESVPVLRNSKEKTEEMLMQSEKRKQLLKTESIKESESLKELVQPEIQPQEFLNRRYMTEVNFSRKQENEEQSWEADYARKPGLLKCWNTLPEPDGQEKKKESLESWESSLKSQEVSKPVVSFEQEKLRPTLQEEQKQQISMAPVSQWKPESPKSKVGSPQEEQNVQETPKPWVVQPQKEQDPKKLPPGSWAVSVQSEQSGSRSWTTPVCREQASVQPGTPVSWENNAENQKHSLVPQSQISLKSWGAASAGLLPNDKVPPRKLNVEPKDVPKPMPQPIDSSSPFPKDPALRKEKLQDLMTQIQGTCNFMQESVLDVDTPSSAIPSSQPPSASPVSTVSAEQNLSNQSDFLQEPSKASSPVTCSSNACLVTTDQASLGSETEFMTSETPEMVAPPCKPASALASPNPPLSKGFQLPPASGSSAAISTAPFQAMQTVFNVNAPLPPRKEQAMKESPYSSGYSQSFTSSSTQTVSQCQLPAVHVEQTTQPPETAAGYHPDGTVQVSNGSLAFYPAPTSMFPRPAQPFISSRGALRGCSRGGRLLMNPYRSPGSYKGFDSYRGLPSASSGTYSQLQLQAREYPGTPYSQRDNFQQCYKRSGTSSGLQANSRAGWSDSSQVSSPERDSETFNSGDSGVGDSRSMTPVDVPVTSPAAAILPVHVYPLPQQMRVAFSAARTSNLAPGTLDQPIVFDLLLNNLGETFDLQLGRFNCPVNGTYVFIFHMLKLAVNVPLYVNLMKNEEVLVSAYANDGAPDHETASNHAILQLLQGDKIWLRLHRGAIYGSSWKYSTFSGYLLYQD*

(**c**)

(**d**)

Hypothetical functional domains of the rat Caprin-2 protein, based on alignment to the *Xenopus* RNG105 protein sequence, a paralogue of the well-analyzed rat Caprin 1 protein, highly homologous to Caprin-2.

Solid underline - coiled-coil domain (strong RNA binding)

Dotted underline - nuclear localization signal (NLS)

Double underline - nuclear export signal (NES)

**Wave underline and bold letters** - RGG box (weak RNA binding)
